# On the Cut-Off Value of the Anteroposterior Diameter of the Midbrain Atrophy in Spinocerebellar Ataxia Type 2 Patients

**DOI:** 10.3390/brainsci14010053

**Published:** 2024-01-05

**Authors:** José Alberto Álvarez-Cuesta, Camilo Mora-Batista, Ramón Reyes-Carreto, Frank Jesus Carrillo-Rodes, Sergio J. Torralbaz Fitz, Yanetza González-Zaldivar, Cruz Vargas-De-León

**Affiliations:** 1 Centro de Investigación y Rehabilitación de las Ataxias Hereditarias, VPWP+RM5, Holguín 80100, Cuba; cuesta140560@gmail.com (J.A.Á.-C.); frankjrodes@gmail.com (F.J.C.-R.); gyanetza@gmail.com (Y.G.-Z.); 2 Facultad de Matemáticas, Universidad Autónoma de Guerrero, Chilpancingo de los Bravo 39087, Mexico; 22251526@uagro.mx; 3 University of Miami Leonard M. Miller School of Medicine, Miami, FL 33136, USA; torralbasfitz@gmail.com; 4 División de Investigación, Hospital Juárez de México, Ciudad de México 07760, Mexico; 5 Laboratorio de Modelación Bioestadística para la Salud, Sección de Estudios de Posgrado e Investigación, Escuela Superior de Medicina, Instituto Politécnico Nacional, Ciudad de México 11340, Mexico

**Keywords:** spinocerebellar ataxia, logistic regression, magnetic resonance image, anteroposterior diameter of the midbrain

## Abstract

(1) Background: Spinocerebellar ataxias (SCA) is a term that refers to a group of hereditary ataxias, which are neurological diseases characterized by degeneration of the cells that constitute the cerebellum. Studies suggest that magnetic resonance imaging (MRI) supports diagnoses of ataxias, and linear measurements of the aneteroposterior diameter of the midbrain (ADM) have been investigated using MRI. These measurements correspond to studies in spinocerebellar ataxia type 2 (SCA2) patients and in healthy subjects. Our goal was to obtain the cut-off value for ADM atrophy in SCA2 patients. (2) Methods: This study evaluated 99 participants (66 SCA2 patients and 33 healthy controls). The sample was divided into estimations (80%) and validation (20%) samples. Using the estimation sample, we fitted a logistic model using the ADM and obtained the cut-off value through the inverse of regression. (3) Results: The optimal cut-off value of ADM was found to be 18.21 mm. The area under the curve (AUC) of the atrophy risk score was 0.957 (95% CI: 0.895–0.991). Using this cut-off on the validation sample, we found a sensitivity of 100.00% (95% CI: 76.84%–100.00%) and a specificity of 85.71% (95% CI: 42.13%–99.64%). (4) Conclusions: We obtained a cut-off value that has an excellent discriminatory capacity to identify SCA2 patients.

## 1. Introduction

The group of hereditary cerebellar ataxias is large and exhibits variability in clinical manifestations and heterogeneous genetic origins. Within this group lies autosomal dominant ataxia, commonly referred to as spinocerebellar ataxia (SCA), based on the pattern of atrophy associated with these anatomical regions. Members of this group are identified numerically based on the order in which the locus or gene causing the subtype was identified [1,2,3,4,5,6,7,8]. Currently, approximately 50 genetic subtypes have been described, with the most common being SCA1, SCA2, SCA3, SCA6, SCA7, and dentatorubral-pallidoluysian atrophy (DRPLA).

SCA2 (OMIM 183090) is the second most common autosomal dominant hereditary ataxia worldwide, and Cuba has reported the highest number of patients with this subtype. According to a population study presented by Velázquez-Pérez in 2019 [1,2,3,4,5,6,7,8], in the Holguín province, 95.7% of families with autosomal dominant ataxias belong to the SCA2 molecular form, hence invoking a ‘founder effect’.

The ATXN2 gene is located on the long arm of chromosome 12 (12q23–24.1), and the mutation involves an expanded trinucleotide CAG repeat ranging from 32 to 77 units. The ATXN2 gene encodes for the synthesis of a cytoplasmic protein, ataxin-2, whose function is still unknown, and its synthesis is not restricted to the central nervous system alone; it is also present in many other organs of the body (found in cardiac tissue, kidneys, muscles, lungs, and spleen), accounting for the diverse and varied extracerebellar manifestations. Purkinje neurons exhibit the highest levels of ataxin-2 expression in the central nervous system [1,3,5,6,7,8,9,10,11,12].

The main neuropathological hallmark of SCA2 is early olivopontocerebellar atrophy, accompanied by the degeneration of somatosensory pathways, the thalamus, the substantia nigra, pons, the frontal lobe, the medulla oblongata, cranial nerves, and the anterior horns of the spinal cord, as well as the pallor of the substantia nigra in the midbrain, where a loss of more than 70% of neurons has been reported. From a neuropathological standpoint, the findings indicate that SCA2 represents the most severe form of hereditary ataxias [1,4,5,7,8,12].

Symptoms of the disease in SCA2 typically begin between the third and fourth decades of life, suggesting that carriers of this abnormal gene can remain symptom-free during an initial period of their lives, referred to as the preclinical period of the disease. However, it is known that, during this stage of the disease, various degrees of cerebral atrophy already exist [8,9,11,12,13,14,15,16,17,18,19,20]. The most common manifestations of the disease include cerebellar-origin gait ataxia, dysarthria, dysmetria, adiadochokinesia, and disruptions in muscle stretch reflexes due to hyporeflexia or areflexia in all four limbs [3,5,7,8,9,11,12]. It is noted that the disease progresses very rapidly over 10–15 years due to the neurodegeneration it induces, during which the patient deteriorates physically, mentally, and immunologically due to the current lack of an effective treatment to halt or slow down SCA2 degeneration. Nevertheless, in Cuba at the “Carlos Juan Finlay” Research and Rehabilitation Center for Hereditary Ataxias (CIRAH), through the neurological rehabilitation program, encouraging results have been achieved, slowing down the progressive course of the disease for several years.

Patients with SCA2 are diagnosed based on genetic diagnosis and clinical presentation, where family history is crucial in creating the family tree, allowing for an understanding of the presence of affected individuals in all generations, typical of the autosomal dominant inheritance pattern.

Although the definitive diagnosis of SCAs is through molecular studies that identify the gene and quantify the size of the polyglutamine expansion through horizontal polyacrylamide gel electrophoresis, this test has several limitations in the overall assessment of the disease [1,2,5,10,11,13,14,15,19,21,22].

The information it provides is based only on the presence or absence of the disease-causing gene, making its use as a disease progression biomarker ineffective, although it has been demonstrated that a higher number of CAG repeats are associated with greater severity of the disease in SCA2. Despite the increased availability of genetic tests today, they are often not economically accessible to the patient, especially for the comprehensive genetic study of the entire family nucleus [1,2,10,15,23]. Consequently, a significant portion of the prevalence data for SCAs may be biased. It is estimated that, due to all these limitations, approximately 50% of the prevalence of hereditary ataxias is unknown.

SCAs are neurodegenerative diseases that lead to premature death within a few years and currently lack specific treatment [1,5,7,8,9,10,13,17,24]. Hence, the search for new therapeutics is imperative, especially with advancements in understanding the molecular pathogenesis of many of these disorders. The development of these effective therapies may also be hindered by the heterogeneity of SCAs [1,3,5,6,7,8,9,10,11,12,13], necessitating specific therapeutic approaches for each. However, the emerging treatments on the horizon need to be validated in clinical trials, and a prerequisite for such trials is the need for objective and more precise evaluation tools than the clinical scores currently used to assess disease progression and the response of the drugs being tested (Assessment and Rating of Ataxia (SARA) and the Inventory of Non-Ataxia Symptoms (INAS)).

## 2. Preliminary

The advent of brain imaging studies, which are now considered indispensable, made it possible to demonstrate the atrophy of the cerebellum, brainstem, spinal cord, and brain. This atrophy is even evident in presymptomatic carriers of SCA2 [2,3,13,21,23,24,25,26,27,28,29,30,31,32,33,34,35,36,37,38,39,40,41]. Nuclear magnetic resonance (NMR) imaging is particularly useful due to its excellent tissue discrimination and its superiority over computed axial tomography by eliminating “noise” caused by bony structures in the posterior cranial fossa. NMR provides a substantial correspondence between atrophy patterns shown by images and morphometric alterations of the anatomical structure [2,3,13,21,24,25,26,33]. Many authors [1,3,5,12,18,20] have reported the atrophy present in these SCAs and are attempting to establish prognostic criteria in relation to the degree of involvement of anatomical structures and clinical manifestations of the patient.

In the analysis of existing literature, it was noted that certain semi-quantitative parameters through magnetic resonance imaging (MRI) are useful in objectively and reproducible determining the atrophy of various nervous structures [1,2,3,7,8,10,13,17,18,20,24,25,26,40,42,43,44], especially using volumetric segmentation methods. In this regard, a systematic review was conducted regarding the use of MRI to diagnose and measure cerebral atrophy progression. From the systematic review, it was concluded that diagnosing olivopontocerebellar atrophy in SCA2 patients using quantitative parameters is a current endeavor of researchers but has not yet yielded the expected results [1,2,3,7,8,10,13,16,17,18,20,24,25,26,40,42,43,44,45]. This is due to the fact that, though it has been more than four decades since the introduction of MRI in medicine and five decades since the discovery of computed axial tomography, the diagnosis of posterior fossa cerebral atrophy is still subjective. The mere description of volumetric reduction of the organs contains within it, through increased subarachnoid space, elements that were already described by computed axial tomography [15,27,28,29,30,31,32,33]. A sufficiently validated threshold of normalcy, where we can diagnose atrophy not solely based on visual observation, has not yet been described objectively. Morphometric MRI research involving volumetry has emphasized describing the wide margin between normality represented by healthy control individuals and patients but has not determined a threshold delineating this relationship. All of these studies are based on the use of powerful magnets (3T and above), excellent image acquisition protocols, and artificial intelligence (AI)-driven software capable of segmenting images with and without human supervision [17,20,21,24,26,33,40,42,43]. Perhaps, with the existing low-field MRI scanners, we could have already obtained this threshold, but as of now, it remains to be determined. Reetz et al. used the measurement of the maximum diameter of the midbrain as a variable, because this is one of the organs most affected by atrophy in this subtype of ataxia [3,21,33,34,35,45]. In their study, an efficient method consisting of manual and linear delineation was proposed. This approach will enable us to robustly diagnose mesencephalic atrophy and subsequently (1) differentiate individuals with the mutation based on the degree of atrophy, (2) assess disease progression, (3) predict the onset of symptoms more accurately in presymptomatic carriers, (4) correlate clinical changes expressed through the SARA and INAS scales with the quantitative magnitude of atrophy, and, most importantly, (5) evaluate the potential effects of future therapies. As is known, clinical scales lack sensitivity for this.

Currently, various techniques derived from NMR are being investigated as potential tools to monitor the progression of neurodegeneration in chronic ataxia. These techniques could serve as potential “surrogate markers” or “biomarkers” in clinical trials for future therapeutic drugs [2,12,13,14,17,24,26,42]. However, these studies have some limitations: (1) Often, the study is heterogeneous, comprising various types of SCAs, (2) the study involves diverse and inadequately described image acquisition protocols that do not allow for standardization, (3) the researchers use software that is not easily applicable across different MRI machine brands and inadequately detailed, especially when involving AI, and (4) a variety of MRI techniques, including high-field equipment, is used, and many of these techniques are only accessible in research centers.

Our study uses the anteroposterior diameter (ADM) since the researched literature reports that it is one of the anatomical structures most affected by atrophy in SCA2 patients, exhibiting a loss of over 70% of neurons. This anatomical structure is easily visible in nuclear magnetic resonance images, even in studies with low-field magnetic resonance machines. For this reason, we aimed to obtain a cut-off value of the ADM in SCA2 patients.

## 3. Materials and Methods

### 3.1. Patients and Methods

A case–control study was conducted with SCA2 patients and controls affiliated at the CIRAH in Holguin, Cuba, from November 2020 to July 2022.

#### 3.1.1. Screening of the Control Group and Patients

The study participants were divided into two groups: a first group of SCA2 patients and a second group consisting of healthy individuals.

Inclusion criteria for patients: Individuals with a genetic and molecular diagnosis of SCA2 who provided informed consent to participate in the study.

Inclusion criteria for the control group: Individuals who provided informed consent to participate in the study.

Exclusion criteria for patients and control group: Individuals presenting any degenerative, psychiatric, tumoral, toxic, immune-mediated, metabolic, or infectious disease affecting the nervous system.

All participants were evaluated by two neurologists dedicated to the diagnosis and monitoring of ataxias at the CIRAH.

For the patients, their medical history, family medical history, onset date of symptoms, duration of the disease, and clinical evaluation of the disease using the SARA scale were assessed.

The clinical assessment toolkit for the disease includes scales such as the SARA and INAS [1,4,11,25,45]. Both are useful in assessing the disease’s status, progression, and neurological deficit. They have been validated and are now widely used for both observational and interventional studies worldwide [4,11,25].

#### 3.1.2. Conducting the Cranial MRI Study

For the MRI study of the brain performed on all participants, a standardized imaging acquisition protocol was established using a Philips device, model Panorama 0.23 T. The protocol included axial, sagittal, and coronal T1-weighted sequences, FFE 3D-24/90, with a slice thickness of 5.5 mm following established anatomical reference lines [3].

#### 3.1.3. Manual Measurement of Specific Anatomical Structures

A manual linear delineation was performed to measure the maximum anteroposterior diameter of the midbrain. The imaging analysis program ‘Imagis’ version 1.13 was utilized as the measurement tool. These measurements were manually obtained by two blinded radiologists to maintain measurement objectivity. In cases of discrepancies, the average value between the two measurements was taken.

In the mid-sagittal slice of the T1 sequence (at the level of the Sylvian aqueduct), a line parallel to the perpendicular or major axis of the brainstem (midbrain, pons, and medulla oblongata) was drawn. Another line was drawn at the level of the superior colliculus, transversely or perpendicular to the previous line, representing the maximum ADM [3,21,33,34,35,45].

### 3.2. Statistical Analysis

The data are presented as the mean (standard deviation, SD) and frequency (percentage) for numerical and categorical variables, respectively. Categorical variables were compared using the chi-square test and numerical variables were compared using a Student’s *t*-test. The total sample was divided into an estimation sample (80%) and a validation sample (20%).

With the estimation sample, a logistic regression model was implemented for ataxia diagnosis using the variable ADM. The goodness-of-fit of the model to the data was verified using the Hosmer–Lemeshow test. Using the inverse logit transformation, we will obtain the equation of the SCA2 atrophy risk score to obtain the prediction probabilities. The receiver operator characteristic (ROC) curve was used to measure the discriminating ability of the prediction probability P(Y|ADM) of the SCA2 atrophy risk score quantified by the area under the curve (AUC). The optimal cut-off point with the highest Youden index was selected. The cut-off value was obtained by evaluating the cut-off probability in the inverse of P(Y|ADM).

The estimation sample was classified as having or not having ataxia using the cut-off value of the SCA2 atrophy risk score. The following measures of classification table accuracy were determined: Sensitivity (Se), Specificity (Sp), Positive Likelihood Ratio (LR+), Negative Likelihood Ratio (LR-), Positive Predictive Value (PPV), Negative Predictive Value (NPV), and Accuracy, with their confidence intervals at 95%. The kappa coefficient and its 95% confidence interval will be determined to quantify the degree of agreement.

The statistical package R, Version 4.2.1, and MedCalc’s diagnostic test evaluation calculator were used [46]. A value of *p* < 0.05 was considered significant.

## 4. Results

### 4.1. Demographics

The sample consisted of two groups: 66 in the SCA2 group and 33 in the healthy control group (Table 1). Twenty-eight of the SCA2 patients (33.76%) and 11 of the healthy controls (28.20%) were men. The SCA2 patients had a mean age of 45.06 (10.25), while subjects in the control group had a mean age of 36.06 (9.14) (p<0.001). The SCA2 patients, according to the SARA scale, were classified into I (n=0), light stadium (n=44), moderate (n=20), and severe (n=2). It is important to note that the four stages of SCA2 refer to presymptomatic, mild, moderate, and severe, respectively. The difference in ADM (16.08 (1.28) vs. 16.08 (1.28)) was statistically significant between groups (p<0.001).

### 4.2. Derivation of SCA2 Atrophy Risk Score

A logistic regression model was fitted to predict atrophy in SCA2 patients using the variable ADM. Table 2 reports the coefficients and their confidence intervals.

We used the inverse logit transformation of the logistic regression model to obtain the SCA2 atrophy risk score equation, which determines the probability of diagnosising atrophy in SCA2 patients. P(Y|ADM) is the probability of experiencing atrophy due to SCA2 given the anteroposterior diameter of the midbrain.

$$P\left(Y|ADM\right)=\frac{e^{\left(35.814-1.972*ADM\right)}}{1+e^{\left(35.814-1.972*ADM\right)}}$$

**Figure 1:**
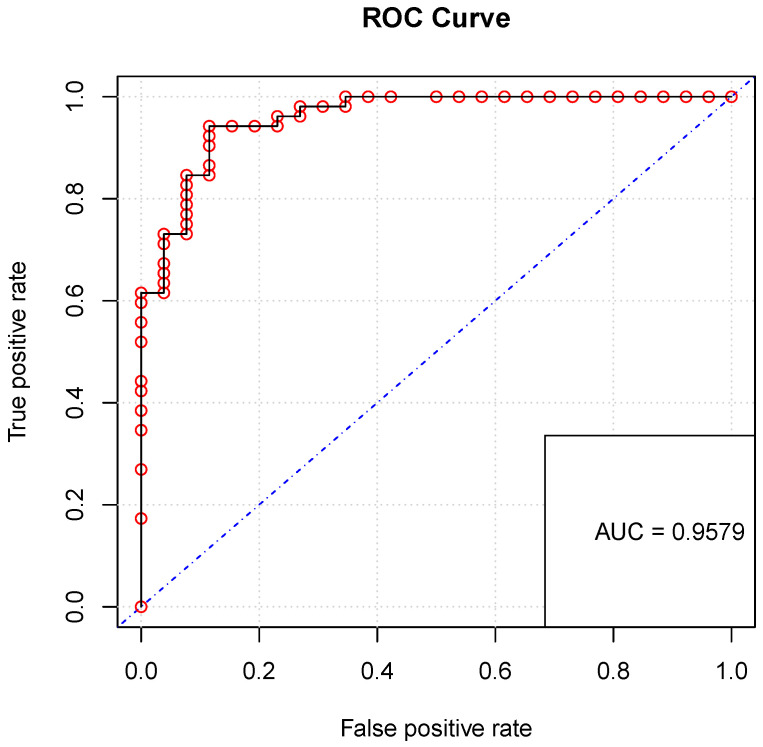
ROC curve of SCA2 atrophy risk score.

An area under the curve of 0.9579 (95% CI: 0.895, 0.991) was obtained, which is a very good predictive atrophy risk score for SCA2 (see Figure 1 of the ROC curve). A cut-off value greater than or equal to 0.477 was determined to diagnose SCA2 as positive with a sensitivity of 94.23 (95% CI 84.05, 98.79), a specificity of 88.46 (95% CI 69.85, 97.55), and a Youden index of 0.826 (95% CI 0.7116, 0.9485). This allowed the study team to determine that the cut-off value for the ADM is 18.21 mm. This value was obtained by evaluating the cut-off probability in the inverse of P(Y|ADM). The value of the Hosmer–Lemeshow statistic is 5.621, which is a measure of how well a model fits the observed data. A *p*-value of 0.689 was obtained, which is the probability of obtaining a Hosmer–Lemeshow statistic that is as extreme as the one observed. This suggests that there is no significant evidence to reject the null hypothesis that the model fits the data adequately.

### 4.3. Validation

Using the cut-off value, the model obtained, and the test sample, a classification matrix was obtained (see Table 3).

The sensitivity and specificity of the atrophy risk score, i.e., 100.00% (95% CI: 76.84% to 100.00%) and 85.71% (95% CI: 42.13% to 99.64%), were found to be good. The positive likelihood ratio, i.e., 7 (95% CI: 1.14 to 42.97), and the negative likelihood ratio, i.e., 0.00%, were also found to be good. The positive (93.33% (95% CI: 69.52% to 98.85%)) and negative (100.00%) predictive values are good. The Kappa value 0.889 (95% CI: 0.678 to 1.000) indicates substantial agreement.

## 5. Discussion and Conclusions

In recent papers, the rarer disorders have often been the subject of qualitative case reports, although there are several recent case–control studies [47,48,49,50,51]. Overall, recent MRI studies have not only provided a better delineation of atrophy patterns in autosomal recessive and dominant ataxias, but have also increasingly moved away from a myopic approach to a cerebellar macrostructure.

In our work, we obtained a cut-off value for ADM, which is useful in the neuroimaging studies of our patients with SCA2, as it guides the genetic study. SCA is diagnosed solely through a genetic study, and there are 48 molecular forms; depending on the molecular form, there will be atrophy in the brain. Observing an ADM value below the threshold in an MRI contributes to the suspicion that the genetic study should focus on SCA2. The fact of having a SCA2 atrophy risk core provides us with a probability regarding the observed measurement, and it will be up to the expert to decide the probability ranges in which the genetic study for SCA2 should be conducted.

Among the MRI studies that have employed manual and linear morphometry in the assessment of olivopontocerebellar atrophy, aiming to identify a reference biomarker for future clinical trials, notable mentions are as follows:

In China, Liang et al. [45] explored the correlation between magnetic resonance imaging characteristics of the brainstem and cerebellum, and the clinical features of spinocerebellar ataxia Type 3/Machado-Joseph disease (SCA3/MJD). They reported a significant inverse correlation between the ADM and the protuberance, as well as the ICARS and SARA scores, and the disease duration when evaluating 32 patients with SCA3/MJD and 36 control subjects. This finding aligns with ours and other researchers regarding the reliability of the ADM for diagnosing cerebral atrophy in SCA3/MJD and other SCAs. However, they did not present the values of this diameter for both research groups.

In a study in Japan by Hara et al. [21], which involved linear and volumetric measurements and manual and linear measurements of ADM, a total of 86 individuals with cerebellar ataxias of various etiologies, both hereditary and non-hereditary, were included. Among them, 34 had SCAs, and only three had SCA2. Interestingly, the ADM in their control group (30 individuals, with no significant variation compared to our sample) measured 15.1 (SD 1.9), considerably lower than our healthy group (39 individuals, measuring 19.19 (SD 1.149)). This difference could be attributed to ethnic and geographical latitude variations. Clearly, the SCA patients had significantly lower values, although these were not considered because the sample was not homogenized for SCA2. Hara et al. show that there is a significant correlation between the ADM and the total ICARS scores. This result motivated our research team to study atrophy in the ADM.

In a Cuban-German collaboration, Reetz et al. [3] utilized volumetry to detect structural changes in the brainstem and cerebellum in various stages of the disease. They involved carriers of the preclinical mutation and patients with SCA2, comparing them with controls. The volumetric assessment of the MRI images confirmed the atrophy of the brainstem and cerebellum characteristic of the neuropathological alterations in the disease for both preclinical mutation carriers and SCA2 patients. However, at that time, the authors did not focus on establishing normal parameters for the volumes of the studied anatomical structures, despite this being the first and largest study comparing carriers and patients with SCA2 to controls.

In order to establish normal ranges for the ADM in healthy Nepalese individuals, correlating the measurement with the age and gender of the patient, Singh et al. (Nepal, 2019) [34] conducted MRI examinations on 103 healthy Nepalese individuals. They found the mean ADM to be 17.048 (SD 0.12) by manual measurements. No statistically significant correlation was identified with the gender of the subjects. Additionally, this diameter slightly decreased after the age of 50 and significantly after 70 years of age. Although the sample was not significant for the Nepalese population, they identified an approximate cut-off point, which closely aligns with the reference values of our cohort, measuring 19.19 (SD 1.149).

However, the performance of image segmentation methods used to date has not yielded the expected results [13,17,20,21,24,26,33,40,42,43]. There are numerous challenges to overcome, particularly the “noise and artifacts” present in MRI (i.e., partial volumes, radiofrequency noise, intensity non-uniformity, degradation, motion, and susceptibility). Since the inception of this research several decades ago, coinciding with the introduction of MRI to medicine, efforts have been made to address these challenges. Over the years, a multitude of segmentation methods have been attempted in the hopes of achieving a simple, precise, reliable, and widely applicable segmentation—essential for current clinical trials.

Additionally, the inherent limitations of images for a single, straightforward, and robust software have posed further difficulties. The heterogeneity and low prevalence of SCAs (1–6 in 100,000) have become another challenge in achieving this goal. Many studies have concluded that their samples are not representative, suggesting a solution to this challenge in the study of hereditary ataxias: the need for multinational studies where cohort sizes align with research needs. In response, initiatives such as the EUROSCA Natural History Study, the Prospective Study of Individuals at Risk for Spinocerebellar Ataxia Types 1, 2, 3, and 6 (RISCA), and the European Friedreich Ataxia Consortium for Translational Studies (EFACTS) have emerged [3,4,14,16,17,52,53,54].

There is no consensus on the use of a unified protocol for obtaining MRI images, nor on the type of MRI machine to be used. Suggestions point towards achieving MRI images with a 1 mm slice thickness, and one challenge given is that this would require the use of high-field MRI machines (1.5–3 T), contingent upon their availability in each country. Hence, a group was formed: Consensus Recommendations by the Ataxia Global Initiative Working Group on MRI Biomarkers, Gülin, Öz. et al. (2023) [39]. They recommend a basic morphometric MRI protocol for use in research and trials, which could allow images obtained in different countries to be used collectively for a common purpose. This goal could be challenging to achieve. Firstly, high-field MRI machines are currently lacking in many geographical locations, and, even if they are available, they must adhere to recommendations. Secondly, even if such a machine were available, the distance patients would need to travel to reach it would pose another limitation, given the wide geographical distribution, even within the same country. This is compounded by the physical and mental toll the disease imposes on these patients, not to mention the economic aspect associated with this.

Conventional MRI is part of the diagnostic workup in the ataxias to confirm cerebellar atrophy. In addition, quantitative MRI technologies have been used to assess structure, connectivity, function, and biochemistry in the ataxias for more than two decades [13,17,55]. A critical need has now emerged for non-invasive and validated biomarkers of brain and cerebellar disease to facilitate upcoming clinical trials in patients with SCAs and more specifically SCA2 [1].

Quantitative neuroimaging research in autosomal recessive ataxias has largely focused on Friedreich’s ataxia (FRDA) and the more common ataxias SCA1, SCA2, SCA3, SCA6, and SCA7. This is because having a large sample of data is difficult due to the low prevalence rate of SCAs. There are studies that have small samples and widely vary in SCA type [13,38,43,56,57,58,59]. However, in the present article, the studied sample consists of SCA2 patients, which can contribute to the quantitative study of atrophy in neuroimaging and more specifically the MRI obtained from SCA2 patients.

There are several limitations of this study. First, we focused solely on measuring the ADM when we could have measured other variables within the olivopontocerebellar axis. Second, we did not include individuals in the preclinical stage of the disease, a decision made by the researchers to establish a broader range between the healthy and the pathological. Future studies are addressing these aspects. Third, the study was not longitudinal.

This study obtained an atrophy risk score from a considerable sample of SCA2 patients and healthy controls. The atrophy risk score has a predictive ability of 95.7% on the diagnosis of ADM atrophy in SCA2 patients, making it a valuable result in the study of SCAs. It may be the first study of this type, based on a comprehensive literature search. The results may help in the search for effective treatments for the rehabilitation of these patients and in the improvement of their quality of life.

Moreover, we reported a threshold value of 18.21 mm in the ADM, which indicates that atrophy due to SCA2 may be present below this value. It is important to emphasize that having atrophy in the ADM below this threshold does not imply that an individual suffers from SCA2. This disease is only diagnosed by a genetic study. The contribution is useful for assessing whether a screening test for SCA2 should be conducted.

## Figures and Tables

**Table 1:** Patient characteristics and anteroposterior diameter of the midbrain.

Feature	SCA2 Patients	Control Patients	*p*-Value
	*n* = 66	*n* = 33	
Sex (Male) ^1^	28 (33.73)	11 (28.20)	0.15128
Age (Years) ^2^	45.06 (10.25)	36.06 (9.14)	<0.0001
SARA scale ^1^			
Light Stadium (13–14)	44 (66.7)	NA	NA
Stage Moderate (15–26)	20 (30.3)	NA	NA
Severe stage (27–34)	2 (3.0)	NA	NA
ADM ^2^	16.08 (1.28)	19.13 (0.94)	<0.001

^1^ Absolute frequency (*n*) and percentage (%). ^2^ Mean (SD); SD, Standard Deviation.

**Table 2:** Coefficient of the model with confidence intervals.

	B	ES	95% CI	*p*-Value
Intercept	35.814	8.358	(22.212, 55.659)	<0.001
ADM	−1.972	0.461	(−3.067, −1.219)	<0.001

**B**: regression coefficient; **ES**: error standard; **95% CI**: 95% confidence interval.

**Table 3:** Classification matrix for SCA2.

		Diagnosis of Spinocerebellar Ataxia Type 2	
		Present	Absent
**SCA2 atrophy risk score**	Positive	14	1
	Negative	0	6

## Data Availability

Data are contained within the article.

## Abbreviations

The following abbreviations are used in this manuscript:

SCA	Spinocerebellar Ataxias
MRI	Magnetic Resonance Image
ADM	Anteroposterior Diameter of the Midbrain
MJD	Machado-Joseph Disease
CIRAH	Centro para la Investigación y Rehabilitación de Ataxias Hereditarias
CONAHCYT	Consejo Nacional de Humanidades, Ciencias y Tecnologías

## References

[1] Klockgether T., Mariotti C., Paulson H.L. (2019). Spinocerebellar ataxia. Nat. Rev. Dis. Prim..

[2] Klaes A., Reckziegel E., Franca M.C., Rezende T.J.R., Vedolin L.M., Jardim L.B., Saute J.A. (2016). MR Imaging in Spinocerebellar Ataxias: A Systematic Review. Am. J. Neuroradiol..

[3] Reetz K., Rodríguez-Labrada R., Dogan I., Mirzazade S., Romanzetti S., Schulz J.B., Cruz-Rivas E.M., Alvarez-Cuesta J.A., Aguilera Rodríguez R., Gonzalez Zaldivar Y. (2018). Brain atrophy measures in preclinical and manifest spinocerebellar ataxia type 2. Ann. Clin. Transl. Neurol..

[4] Velázquez-Pérez L., Medrano-Montero J., Rodríguez-Labrada R., Canales-Ochoa N., Campins Alí J., Carrillo Rodes F.J., Rodríguez Graña T., Hernández Oliver M.O., Aguilera Rodríguez R., Domínguez Barrios Y. (2020). Hereditary Ataxias in Cuba: A Nationwide Epidemiological and Clinical Study in 1001 Patients. Cerebellum.

[5] Velázquez-Pérez L.C., Rodríguez-Labrada R., Fernandez-Ruiz J. (2017). Spinocerebellar Ataxia Type 2: Clinicogenetic Aspects, Mechanistic Insights, and Management Approaches. Front. Neurol..

[6] Rodríguez-Labrada R., Martins A.C., Magaña J.J., Vazquez-Mojena Y., Medrano-Montero J., Fernandez-Ruíz J., Cisneros B., Teive H., McFarland K.N., Saraiva-Pereira M.L. (2020). Founder Effects of Spinocerebellar Ataxias in the American Continents and the Caribbean. Cerebellum.

[7] Paulson H.L., Shakkottai V.G., Clark H.B., Orr H.T. (2017). Polyglutamine spinocerebellar ataxias—From genes to potential treatments. Nat. Rev. Neurosci..

[8] Jung B.C., Choi S.I., Du A.X., Cuzzocreo J.L., Ying H.S., Landman B.A., Perlman S.L., Baloh R.W., Zee D.S., Toga A.W. (2012). MRI Shows a Region-Specific Pattern of Atrophy in Spinocerebellar Ataxia Type 2. Cerebellum.

[9] Pilotto F., Saxena S. (2018). Epidemiology of inherited cerebellar ataxias and challenges in clinical research. Clin. Transl. Neurosci..

[10] Perlman S. (1993–2023). Hereditary Ataxia Overview.

[11] Moriarty A., Cook A., Hunt H., Adams M.E., Cipolotti L., Giunti P. (2016). A longitudinal investigation into cognition and disease progression in spinocerebellar ataxia types 1, 2, 3, 6, and 7. Orphanet J. Rare Dis..

[12] Meira A.T., Arruda W.O., Ono S.E., de Carvalho Neto A., Raskin S., Camargo C.H.F., Teive H.A.G. (2019). Neuroradiological Findings in the Spinocerebellar Ataxias. Tremor Other Hyperkinetic Movements.

[13] Ashizawa T., Öz G., Paulson H.L. (2018). Author Correction: Spinocerebellar ataxias: Prospects and challenges for therapy development. Nat. Rev. Neurol..

[14] Mascalchi M., Vella A. (2020). Neuroimaging Biomarkers in SCA2 Gene Carriers. Int. J. Mol. Sci..

[15] Kim D.H., Kim R., Lee J.Y., Lee K.M. (2021). Clinical, Imaging, and Laboratory Markers of Premanifest Spinocerebellar Ataxia 1, 2, 3, and 6: A Systematic Review. J. Clin Neurol..

[16] Wilke C., Haas E., Reetz K., Faber J., Garcia-Moreno H., Santana M.M., van de Warrenburg B., Hengel H., Lima M., Filla A. (2020). Neurofilaments in spinocerebellar ataxia type 3: Blood biomarkers at the preataxic and ataxic stage in humans and mice. EMBO Mol. Med..

[17] Öz G., Harding I.H., Krahe J., Reetz K. (2020). MR imaging and spectroscopy in degenerative ataxias: Toward multimodal, multisite, multistage monitoring of neurodegeneration. Curr. Opin. Neurol..

[18] Monte T.L., Reckziegel E.d.R., Augustin M.C., Locks-Coelho L.D., Santos A.S.P., Furtado G.V., de Mattos E.P., Pedroso J.L., Barsottini O.P., Vargas F.R. (2018). The progression rate of spinocerebellar ataxia type 2 changes with stage of disease. Orphanet J. Rare Dis..

[19] Silva R.N.d., Vallortigara J., Greenfield J., Hunt B., Giunti P., Hadjivassiliou M. (2019). Diagnosis and management of progressive ataxia in adults. Pract. Neurol..

[20] Mascalchi M., Diciotti S., Giannelli M., Ginestroni A., Soricelli A., Nicolai E., Aiello M., Tessa C., Galli L., Dotti M.T. (2014). Progression of Brain Atrophy in Spinocerebellar Ataxia Type 2: A Longitudinal Tensor-Based Morphometry Study. PLoS ONE.

[21] Hara D., Maki F., Tanaka S., Sasaki R., Hasegawa Y. (2016). MRI-based cerebellar volume measurements correlate with the International Cooperative Ataxia Rating Scale score in patients with spinocerebellar degeneration or multiple system atrophy. Cerebellum Ataxias.

[22] Cocozza S., Pontillo G., De Michele G., Di Stasi M., Guerriero E., Perillo T., Pane C., De Rosa A., Ugga L., Brunetti A. (2021). Conventional MRI findings in hereditary degenerative ataxias: A pictorial review. Neuroradiology.

[23] Coarelli G., Brice A., Durr A. (2018). Recent Advances in Understanding Dominant Spinocerebellar Ataxias from Clinical and Genetic Points of View. F1000Research.

[24] Straub S., Mangesius S., Emmerich J., Indelicato E., Nachbauer W., Degenhardt K.S., Ladd M.E., Boesch S., Gizewski E.R. (2020). Toward quantitative neuroimaging biomarkers for Friedreich’s ataxia at 7 Tesla: Susceptibility mapping, diffusion imaging, R2 and R1 relaxometry. J. Neurosci. Res..

[25] Kim M., Ahn J.H., Cho Y., Kim J.S., Youn J., Cho J.W. (2019). Differential value of brain magnetic resonance imaging in multiple system atrophy cerebellar phenotype and spinocerebellar ataxias. Sci. Rep..

[26] Adanyeguh I.M., Perlbarg V., Henry P.G., Rinaldi D., Petit E., Valabregue R., Brice A., Durr A., Mochel F. (2018). Autosomal dominant cerebellar ataxias: Imaging biomarkers with high effect sizes. NeuroImage Clin..

[27] Lee S.H., Altamarino L.S., Toglia J.U. (1978). Cerebellar atrophy: Pneumoencephalography and computerized tomography correlation. Neuroradiology.

[28] Steele J.R., Hoffman J. (1981). Brainstem evaluation with CT cisternography. Am. J. Roentgenol..

[29] Abe S., Miyasaka K., Tashiro K., Takei H., Isu T., Tsuru M. (1983). Evaluation of the Brainstem with High-Resolution CT in Cerebellar Atrophic Processes. AJNR Am. J. Neuroradiol..

[30] Koller W.C., Glatt S.L., Perlik S., Huckman M.S., Fox J.H. (1981). Cerebellar atrophy demonstrated by computed tomography. Neurology.

[31] Allen J.H., True Martin J., William McLain L. (1979). Computed Tomography in Cerebellar Atrophic Processes. Radiology.

[32] Ramos A., Quintana F., Díez C., Leno C., Berciano J. (1987). CT Findings in Spinocerebellar Degeneration. AJNR Am. J. Neuroradiol..

[33] Kumar S., Chand R., Gururaj A., Jeans W. (1995). CT Features of Olivopontocerebellar Atrophy in Children. Acta Radiol..

[34] Singh S., Sharma B.R., Bhatta M., Poudel N. (2019). Measurement of Anteroposterior diameters of normal brainstem by Magnetic Resonance Imaging. J. Gandaki Med-Coll.-Nepal.

[35] Metwally M.I., Basha M.A.A., AbdelHamid G.A., Nada M.G., Ali R.R., frere R.A.F., Elshetry A.S.F. (2021). Neuroanatomical MRI study: Reference values for the measurements of brainstem, cerebellar vermis, and peduncles. Br. J. Radiol..

[36] Martínez Guerrero J., Paz-Gutiérrez J., Vega-Gaxiola S.B. (2016). Ataxia Espinocerebelosa Tipo 2. Arch. Neurociencias.

[37] Jandeaux C., Kuchcinski G., Ternynck C., Riquet A., Leclerc X., Pruvo J.P., Soto-Ares G. (2019). Biometry of the Cerebellar Vermis and Brain Stem in Children: MR Imaging Reference Data from Measurements in 718 Children. AJNR Am. J. Neuroradiol..

[38] Cuesta J., Batista C., Vargas-De-León C., Rodes F., Guzman-Martinez M., Fitz S., Benedicenti L., Liu Z. (2022). Search for A Midbrain Anteroposterior Diameter Threshold to Study Brain Atrophy in Spinocerebellar Ataxia Type 2. Proceedings of the 8th World Congress on Electrical Engineering and Computer Systems and Science, EECSS 2022.

[39] Öz G., Cocozza S., Henry P., Lenglet C., Deistung A., Faber J., Schwarz A., Timmann D., Van Dijk K., Harding I. (2023). MR Imaging in Ataxias: Consensus Recommendations by the Ataxia Global Initiative Working Group on MRI Biomarkers. Cerebellum.

[40] Ye C., Yang Z., Ying S.H., Prince J.L. (2015). Segmentation of the Cerebellar Peduncles Using a Random Forest Classifier and a Multi-object Geometric Deformable Model: Application to Spinocerebellar Ataxia Type 6. Neuroinformatics.

[41] Cabeza-Ruiz R., Velázquez-Pérez L., Linares-Barranco A., Pérez-Rodríguez R. (2022). Convolutional Neural Networks for Segmenting Cerebellar Fissures from Magnetic Resonance Imaging. Sensors.

[42] Ravanfar P., Loi S.M., Syeda W.T., Van Rheenen T.E., Bush A.I., Desmond P., Cropley V.L., Lane D.J.R., Opazo C.M., Moffat B.A. (2021). Systematic Review: Quantitative Susceptibility Mapping (QSM) of Brain Iron Profile in Neurodegenerative Diseases. Front. Neurosci..

[43] Xie F., Weihua L., Lirong O., Wang X., Xing W. (2020). Quantitative susceptibility mapping in spinocerebellar ataxia type 3/Machado–Joseph disease (SCA3/MJD). Acta Radiol..

[44] Coarelli G., Darios F., Petit E., Dorgham K., Adanyeguh I., Petit E., Brice A., Mochel F., Durr A. (2021). Plasma neurofilament light chain predicts cerebellar atrophy and clinical progression in spinocerebellar ataxia. Neurobiol. Dis..

[45] Liang X., Jiang H., Chen C., Zhou G., Wang J., Zhang S., Lei L., Wang X., Tang B. (2009). The correlation between magnetic resonance imaging features of the brainstem and cerebellum and clinical features of spinocerebellar ataxia 3/Machado-Joseph disease. Neurol. India.

[46] MedCalc Software Ltd. (2023). Diagnostic Test Evaluation Calculator. Version 22.014. https://www.medcalc.org/calc/diagnostic_test.php.

[47] Selvadurai L.P., Corben L.A., Delatycki M.B., Storey E., Egan G.F., Georgiou-Karistianis N., Harding I.H. (2020). Multiple mechanisms underpin cerebral and cerebellar white matter deficits in Friedreich ataxia: The IMAGE-FRDA study. Hum. Brain Mapp..

[48] Yang Y., Wang S., Zeng N., Duan W., Chen Z., Liu Y., Li W., Guo Y., Chen H., Li X. (2022). Lung Radiomics Features Selection for COPD Stage Classification Based on Auto-Metric Graph Neural Network. Diagnostics.

[49] Yang Z.h., Shi C.h., Zhou L.n., Li Y.s., Yang J., Liu Y.t., Mao C.y., Luo H.y., Xu G.w., Xu Y.m. (2019). Metabolic Profiling Reveals Biochemical Pathways and Potential Biomarkers of Spinocerebellar Ataxia 3. Front. Mol. Neurosci..

[50] Yap K.H., Abdul Manan H., Yahya N., Azmin S., Mohamed Mukari S.A., Mohamed Ibrahim N. (2022). Magnetic Resonance Imaging and Its Clinical Correlation in Spinocerebellar Ataxia Type 3: A Systematic Review. Front. Neurosci..

[51] Rezende T.J.R., Martinez A.R.M., Faber I., Girotto Takazaki K.A., Martins M.P., de Lima F.D., Lopes-Cendes I., Cendes F., França M.C. (2019). Developmental and neurodegenerative damage in Friedreich’s ataxia. Eur. J. Neurol..

[52] Mascalchi M., Vella A., Politis M. (2018). Chapter Four-Neuroimaging Applications in Chronic Ataxias. Imaging in Movement Disorders: Imaging Applications in Non-Parkinsonian and Other Movement Disorders.

[53] Garali I., Adanyeguh I.M., Ichou F., Perlbarg V., Seyer A., Colsch B., Moszer I., Guillemot V., Durr A., Mochel F. (2018). A strategy for multimodal data integration: Application to biomarkers identification in spinocerebellar ataxia. Briefings Bioinform..

[54] Buijsen R.A., Toonen L.J., Gardiner S.L., van Roon-Mom W.M. (2019). Genetics, Mechanisms, and Therapeutic Progress in Polyglutamine Spinocerebellar Ataxias. Neurotherapeutics.

[55] Dogan I., Romanzetti S., Didszun C., Mirzazade S., Timmann D., Saft C., Schöls L., Synofzik M., Giordano I.A., Klockgether T. (2019). Structural characteristics of the central nervous system in Friedreich ataxia: An in vivo spinal cord and brain MRI study. J. Neurol. Neurosurg. Psychiatry.

[56] Faber J., Schaprian T., Berkan K., Reetz K., França M.C., de Rezende T.J.R., Hong J., Liao W., van de Warrenburg B., van Gaalen J. (2021). Regional Brain and Spinal Cord Volume Loss in Spinocerebellar Ataxia Type 3. Mov. Disord. Off. J. Mov. Disord. Soc..

[57] Brockmann K., Reimold M., Globas C., Hauser T.K., Walter U., Machulla H.J., Rolfs A., Schöls L. (2012). PET and MRI reveal early evidence of neurodegeneration in spinocerebellar ataxia type 17. J. Nucl. Med. Off. Publ. Soc. Nucl. Med..

[58] Lindig T., Bender B., Kumar V.J., Hauser T.K., Grodd W., Brendel B., Just J., Synofzik M., Klose U., Scheffler K. (2019). Pattern of Cerebellar Atrophy in Friedreich’s Ataxia-Using the SUIT Template. Cerebellum.

[59] Li Q.F., Dong Y., Yang L., Xie J.J., Ma Y., Du Y.C., Cheng H.L., Ni W., Wu Z.Y. (2019). Neurofilament light chain is a promising serum biomarker in spinocerebellar ataxia type 3. Mol. Neurodegener..

